# The midcortical-line is more reliable than the T-line in predicting stem anteversion in patients with developmental hip dysplasia after total hip arthroplasty

**DOI:** 10.3389/fsurg.2022.966617

**Published:** 2022-09-01

**Authors:** Ziang Jiang, Rongshan Cheng, Willem Alexander Kernkamp, Chunjie Xia, Junjie Liang, Liao Wang, Tsung-Yuan Tsai

**Affiliations:** ^1^ School of Biomedical Engineering and Med-X Research Institute, Shanghai Jiao Tong University, Shanghai, China; ^2^ Department of Orthopaedic Surgery, Shanghai Ninth People's Hospital, Jiao Tong University School of Medicine, Shanghai, China; ^3^ Engineering Research Center of Digital Medicine and Clinical Translation, Ministry of Education, Shanghai, China; ^4^ Department of Orthopaedic Surgery, Leiden University Medical Center, Leiden, Netherlands; ^5^ School of Mechanical Engineering, Shanghai Jiao Tong University, Shanghai, China; ^6^ Guangxi Clinical Research Center for Digital Medicine and 3D Printing, Guigang City People's Hospital, Guigang, China

**Keywords:** midcortical-line, T-line, postoperative stem anteversion, developmental dysplasia of the hip (DDH), total hip arthroplasty (THA)

## Abstract

**Background:**

Precise preoperative planning improves postoperative outcomes in total hip arthroplasty (THA), especially in developmental dysplasia of the hip (DDH) cases. Previous studies used the T-line and midcortical-line as preoperative landmarks to predict postoperative stem anteversion (PSA). However, the most reliable landmark in predicting PSA in DDH patients remains unclear. To find the most reliable measurement to predict the PSA in DDH patients, this study compared the midcortical-line and T-line at different femoral neck levels.

**Methods:**

Pre- and postoperative Computed Tomography (CT) scans of 28 hips in 21 DDH patients who received THA were obtained for three-dimensional femoral models. The preoperative CT scan was used to measure the anteversion of the midcortical-line on the axial cross-sectional plane images (AM-CT), the anteversion of the midcortical-line from 3D models (AM-3D), and the T-line from 3D models (AT-3D) at simulated osteotomy planes at 5 and 10 mm heights proximal to the base of the lesser trochanter. The correlation between the preoperative femoral anteversion (AM-CT, AM-3D, AT-3D) and the PSA was assessed to evaluate the prediction accuracy.

**Results:**

The correlations between the AM-CT and the PSA were 0.86 (mean difference (MD) = 1.9°) and 0.92 (MD = −3.0°) at 5 and 10 mm heights, respectively. The correlation between the AM-3D and the PSA were 0.71 (MD = −11.6°) and 0.61 (MD = −12.9°) at 5 and 10 mm heights. The AT-3D was significantly greater (MD = 15.4°) than the PSA (*p*-value <0.01) at 5 mm cutting height, and the correlation between the AT-3D and the PSA was 0.57 (MD = 7.8°) at 10 mm cutting height.

**Conclusions:**

The AM-CT at the 10 mm height had the strongest correlation with the PSA and was more reliable in predicting the PSA when compared with the AM-3D and the AT-3D in DDH patients.

## Introduction

Appropriate postoperative stem anteversion (PSA) in total hip arthroplasty (THA) is critical to achieving implant stability, the optimal range of motion (ROM), and avoiding impingement (1–8). The combined anteversion technique, which considers the sum of acetabular cup anteversion and femoral anteversion, has been clinically proven to prevent implant impingement if controlled in a safe zone of 25°–50° (8–11). The stem anteversion is more challenging to control during surgery, or to predict preoperatively. Therefore, the “femur-first” technique (9, 12) was developed. The cup anteversion can then be intraoperatively calculated to the stem anteversion and match the safe zone. Therefore, the prediction of PSA can play a decisive role in the implantation target of the acetabular cup, which can optimize the combined anteversion of the preoperative planning process. Accurate predictions of the PSA may improve surgical outcomes after THA.

Few studies have used the anatomical landmarks of medical images to predict the PSA prior to THA implantation. Suh et al. (13) reported that the midcortical-line is compatible with the true femoral anteversion using a single slice of CT. However, Tsukeoka et al. (14, 15) demonstrated that the stem tended to retroversion compared with the midcortical-line on the cut surface of the femoral neck. These differences in the reliability of the midcortical-line could attribute to different methodologies. Others used the lesser trochanter to estimate the PSA intraoperatively which has shown to have a mean difference of <5° (16). However, it is difficult to evaluate the version of the lesser trochanter using a surgical incision other than the posterior approach. The T-line landmark that connects the trochanteric fossa and the inferior margins on the cut surface was found to be a reliable and reproducible intraoperative reference to produce a stable and functional THA (15). During THA surgery, the T-line reproduces the native femoral anteversion (NFA) in osteoarthritis and DDH patients (14, 15). Through the definition of femoral stem torsion in THA simulation surgery, the T-line was also validated that the accuracy of using a T-line to guide version during implantation of the femoral stem would not be affected by the degree of subluxation of the femoral head (15). 3D analyzes are thought to better simulate the intraoperative view before THA. Detailed 3D information may be particularly helpful in complex preoperative planning in patients with developmental dysplasia of the hip (DDH) (5, 14, 15, 17). However, 3D analysis requires intensive work and is therefore not practical in daily practice. The relatively convenient use of CT images for PSA prediction has also not been evaluated for its efficiency in DDH patients.

Furthermore, the level of the CT images selected in predicting the PSA also affected the accuracy. Yu et al. (3) showed that AM-CT, which selected the CT images at a proximal level, accurately predicted PSA for Crowe type II/III DDH patients with a posterolateral approach and “acetabular-first” technique. Tsukeoka et al. (15) simulated the osteotomy process on 3D femoral models, which showed that the AT-3D at 5 mm cutting height proximal to the lesser trochanter reproduced the NFA better than that at 10 mm. However, the effect of different osteotomy levels using the T-line measurement on predicting the PSA for DDH patients remains unknown, and the confirmation of the optimal femoral neck level requires further investigation.

This study aimed to investigate: (1) whether the midcortical-line or the T-line was more reliable in predicting the PSA in DDH patients; (2) to find the optimal femoral neck level at which the T-line and midcortical-line could better predict PSA in DDH patients.

## Materials and methods

### Patient demographics

The Internal Review Board approved this study. 28 hips were enrolled in this study retrospectively. The inclusion criteria were: patients with DDH Crowe grade I to IV who had undergone cementless THA and had received pre-and postoperative femoral CT scans between May 2013 and September 2015. The exclusion criteria were: patients who underwent an osteotomy lower than the lesser trochanter level during surgery, patients without femoral head or neck, patients who had prior hip surgery, patients who missed the pre-operative or postoperative lower limb CT images, and patients who had a surgical complication of dislocation or component subluxation on the implanted hips. A total of 17 hips were Crowe type I (<50% subluxation); 6 Crowe type II/III (50%–75%/75%–100% subluxation); and 5 Crowe type IV (>100% subluxation) (18) (Table 1). According to the guideline of Crowe classification, the dysplasia with the lateral center-edge angle (LCEA) of the participants was less than 20° measured from a radiograph.

**Table 1:** Characteristics in DDH patients.

Parameters	Mean ± SD
Age	64.5 ± 8.9
Weight (kg)	64.0 ± 10.2
Height (m)	1.6 ± 0.1
BMI (kg/m^2^)	24.7 ± 3.1

### Surgical procedure

All DDH hips received cementless THA prostheses (Stryker Secur-Fit, Mahwah, New Jersey, United States; DePuy SUMMIT, Warsaw, IN, United States) with metaphyseal fit stems by the same experienced arthroplasty surgeon (ZZ) using a posterolateral approach. Intraoperatively, the femoral stem was implanted using the “femur-first” technique (9, 12), in combination with the evaluation of the medullary cavity, femoral geometry, and acetabular position (19), and experience.

### Measurements based on CTs

**Figure 1:**
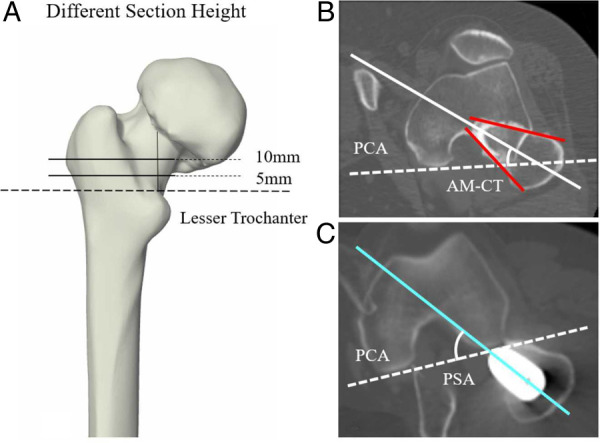
The schematic illustration of the measurements based on CT. (**A**) The selection of two different CT section height on femur, which are 5 mm and 10 mm height above the proximal end of the lesser trochanter. (**B**) AM-CT was defined as the angle between the PCA (white dotted line) and the midcortical-line (white solid line), which is the angular bisector of anterior cortex and posterior cortex (red solid line). (**C**) PSA was defined as the angle between the PCA and the femoral stem neck axis (bright sky-blue solid line).

The pre- and postoperative CT scans were obtained using 128-slices CT scanners (Somatom Definition Flash®, Siemens Healthcare, Germany) with 1 mm slice thickness and in-plane resolution of 0.98 mm. The preoperative CT images at 5 mm and 10 mm heights above the lesser trochanter were selected (Figure 1A). The midcortical-line was defined as the anterior and posterior cortex's angular bisector (3, 13). The AM-CT is the angle between the midcortical-line and Posterior Condylar Axis (PCA) on each level as proposed (3) (Figure 1B). The PSA was measured as the angle between the femoral stem neck axis on the axial CT images and the PCA from the postoperative CTs (Figure 1C). The anatomical coordinate system referred to the International Society of Biomechanics (ISB) recommendations (20).

### Measurements based on 3D models

**Figure 2:**
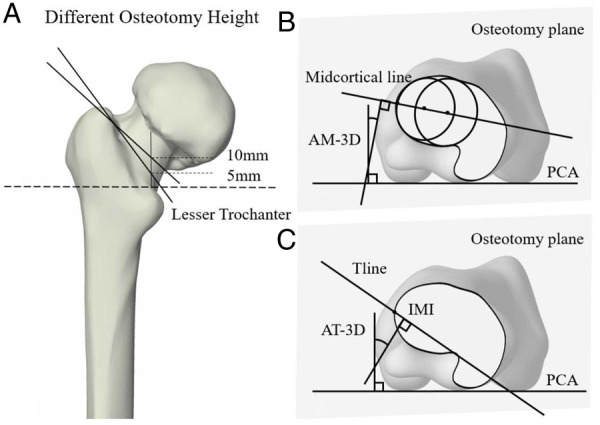
The schematic illustration of the measurements based on 3D model. (**A**) The simulated osteotomy plane pass through the center of piriformis fossa and the location at the 5 mm and 10 mm heights above the lesser trochanter. (**B**) AM-3D was defined as the angle between the midcortical-line and the PCA. (**C**) AT-3D was defined as the angle between the PCA and the T-line. IMI was the intersection of midcortical-line with the inferior margins of the osteotomy plane.

The pre- and postoperative CT images were imported into commercial software (Amira 6.7, Thermo Fisher Scientific, Waltham, MA, United States) to reconstruct the 3D surface models (21). The anteversion using the midcortical-line and T-line were measured based on the 3D models (AM-3D; AT-3D) (14). The simulated osteotomy plane was determined through the center of the piriformis fossa and the 5 and 10 mm cutting heights proximal to the lesser trochanter (Figure 2A). AM-3D was defined as the angle between the midcortical-line (the connecting line between the center of the best fitting circles obtained from the medial and inferior margins of the simulated osteotomy plane) and the PCA (14) (Figure 2B). AT-3D was defined as the angle between the T-line (the line connecting the trochanteric fossa and the inferior margins of the plane) and the PCA (14) (Figure 2C).

### Statistical analysis

All data met the normal distribution, and the measurements were calculated using Excel 2016 (Microsoft Excel, Redmond, WA, United States). The data were analyzed using the mean values ± standard deviation of PSA, AM-CT, AM-3D, and AT-3D. The differences between AM-CT, AM-3D, AT-3D, and PSA were calculated by the mean difference (MD). Interobserver and intraobserver reliability of the measurements was estimated by the intraclass correlation coefficient (ICC). Pearson correlation coefficients (*r*) were used to evaluate the correlations between AM-CT, AM-3D, AT-3D, and PSA. The student's *t*-test was used to determine differences between parameters at 5 and 10 mm heights. The statistical significance level (*α*) was set at 0.05.

## Results

The ICC for intra-observer and interobserver reliabilities were >0.9 for all measurements. The two-tailed Pearson correlation coefficients are statistically significant for all measures.

A strong correlation was found for the AM-CT and the PSA at the 5 and 10 mm heights (respectively, *r* = 0.86 with *p*-value = 0.000 and *r* = 0.92 with *p*-value = 0.000), and no statistically significant difference was found (*p*-value = 0.662 and 0.495). The mean difference (MD) between the AM-CT at 5 mm height (31.8° ± 15.3°) and the PSA (29.8° ± 17.7°) was 1.9° ± 8.8°, and the MD between the AM-CT at 10 mm height (26.8° ± 14.9°) and PSA was −3.0° ± 7.1° (Table 2).

**Table 2:** The different anteversions simulated based on various reference landmarks.

Parameters	Height (mm)	Angle (°)^a^	Difference (°)^a^	*t*-test *p*-value	*r*	Pearson correlation *p*-value
AM-CT	5	31.8 ± 15.3	1.9 ± 8.8	0.662	0.86	0.000**
	10	26.8 ± 14.9	−3.0 ± 7.1	0.495	0.92	0.000**
AM-3D	5	18.3 ± 12.5	−11.6 ± 12.5	0.007*	0.71	0.000**
	10	16.9 ± 12.3	−12.9 ± 14.2	0.003*	0.61	0.001**
AT-3D	5	45.2 ± 17.5	15.4 ± 16.5	0.002*	0.56	0.001**
	10	37.6 ± 15.2	7.8 ± 15.5	0.084	0.57	0.001**
PSA	N/A	29.8 ± 17.7	N/A	N/A	N/A	N/A

Difference = (AM-CT/AM-3D/AT-3D) − PSA; *t*-test = student's *t*-test; *r* = correlation coefficient.

^a^ Expressed as mean ± standard deviation.

* Indicates the difference is statistically significant in student's *t*-test (*p*-value <0.05).

** Indicates the Pearson correlation coefficient is statistically significant (*p*-value <0.01).

A moderate correlation was found between the AM-3D and the PSA at the 5 and 10 mm cutting heights respectively, *r* = 0.71 (*p*-value = 0.000) and *r* = 0.61 (*p*-value = 0.001). The mean AM-3D was 18.3° ± 12.5° at 5 mm and 16.9° ± 12.3° at 10 mm cutting height, which both were significantly smaller than the PSA (*p*-value = 0.007 and 0.003) (Table 2).

A moderate correlation was also found between the AT-3D and the PSA at 5 and at 10 mm cutting heights, respectively, which were *r* = 0.56 (*p*-value = 0.001) and *r* = 0.57 (*p*-value = 0.001) in 5 and 10 mm groups. The mean AT-3D was 45.2° ± 17.5° and was significantly greater than the PSA (*p*-value <0.010) at 5 mm cutting height. The mean AT-3D was 37.6° ± 15.2° and no significant difference was found between AT-3D and PSA (*p*-value = 0.084) at 10 mm cutting height (Table 2).

## Discussion

The main finding of this study was that midcortical-line had higher accuracy in predicting the PSA compared to the T-line. Second, the 10 mm osteotomy level for AM-CT may best predict the PSA compared to the AM-3D or AT-3D in DDH patients. The AM-CT on the axial CT images at the 10 mm height had the strongest correlation (*r* = 0.92) and the smallest difference with PSA (−3.0° ± 7.1°) compared to the other methods. Therefore, clinical use of AM-CT to predict PSA (4, 8, 10) can best determine the anteversion of the acetabular cup and help to control the combined anteversion in the safe zone.

We found that the prediction of anteversion using the T-line showed a moderate correlation with the PSA (*r* = 0.56 and *r* = 0.57 in the 5 and 10 mm groups) and can even be significantly greater than the PSA (MD reached 15.4° in the 5 mm group). This difference may be explained by the conception of the T-line. The T-line is adjusted to get a larger anteversion compared with the midcortical-line. The adjustment corrected the proximal femoral deformity of DDH patients due to the disease. Therefore, T-line can be a useful intraoperative reference that helps reproduce the NFA as the high correlation with the NFA reported in the article of Tsukeoka et al. (15). The anteversion reference can be extremely meaningful in clinical application for the implantation of prostheses like SROM for DDH patients, especially for patients in severe situations. However, the orientation of the cementless femoral stem in implanting was mainly dependent on the geometric shape of the proximal medullary cavity. The intraoperative twist and press-fit result in a certain pathological anteversion but lead to the deviation from the anteversion of the T-line landmark.

On the other hand, the midcortical-line was strongly correlated with the PSA of DDH patients, which is consistent with the previous studies (3, 13). This may be because the midcortical-line is created between the anterior and posterior cortical line and met an actual axis of femoral anteversion (13), which may influence the orientation of the cementless stem in THA during implanting. Moreover, the patients selected in this study included DDH patients with the posterolateral approach, which expands the application range of the conclusion that the AM-CT could be a reliable landmark for predicting the PSA of DDH patients.

The height of the anatomic landmark is critical to the accuracy of prediction. According to the previous studies (3, 13, 15), 5 and 10 mm heights proximal to the base of the lesser trochanter are commonly chosen for osteotomy, which can preserve bone mass and prevent trochanteric fractures. In this study, we observed a strong correlation between the AM-CT at the two levels (5 and 10 mm height proximal to the lesser trochanter) and the PSA. We found the AM-CT at 10 mm height was better than that at 5 mm for predicting the PSA for DDH patients. These results may be because morphological characteristics of the distal femoral medullary cavity in DDH femurs tend to be more circular or elliptical (6, 22), which created more difficulties in confirming the anterior and posterior cortex. Therefore, 5 mm height proximal to the base of the lesser trochanter of the CT slices may cause a slight deviation in confirming the midcortical-line compared to the 10 mm height group. Moreover, the circinal or elliptical medullary cavity in the distal location can provide a relatively greater adjustive range for stem implantation, which resulted in the difference between PSA and predicted stem anteversion (23). Therefore, the CT images at 10 mm above the proximal end of the lesser trochanter are advised to use in preoperative planning for DDH patients accurately.

The explanation of these phenomena is that the design of cementless femoral stems is mainly based on the medullary cavity morphology according to CT images (24). Therefore, the postoperative anteversion of cementless stems with adaptation in implanting may be relatively consistent with the positional relationship between the stem and the proximal femoral medullary canal observed on CT images. This may also explain why the midcortical-line from cross-sectional CT planes can be better correlated with PSA than the 3D models. Therefore, the AM-CT based on the axis CT images was more appropriate for predicting the PSA than the AM-3D based on the 3D models in the preoperative planning. Furthermore, although using the 3D models can simulate the THA surgical procedure, we did not find other landmarks from the osteotomy planes of the 3D femoral model that have high effectiveness in predicting PSA in this study. Therefore, we believe preoperative planning based on CT images provides a good solution for predicting the PSA.

This study has several limitations. First, the sample size of this study was small. However, the Pearson correlation coefficient is statistically significant at the 0.05 level (two-tailed), which verified the validity of the sample. The sample size of this experiment has certain reliability. Limited by the sample size, it is hard for us to make a sub-group analysis separately according to different Crowe types. Nevertheless, the PSA is mainly depending on the cavity morphology, which shared a similar trend in Crowe I–IV proximal femur in the transverse section (23). Moreover, the selected plane for obtaining the landmarks involved minimal areas that were influenced by excessive deformation caused by DDH with different severity, such as the femoral head and neck. Thus, the difference caused by DDH Crowe types in conclusion should be limited in this research. Second, even though the cementless stem was reported as one of the most extensively used stems in younger patients, the use of only one type of femoral stem in this study was limited to a certain extent (22, 25). The cementless stem design mainly relies on the profile of the femoral medullary cavity based on the CT cross-section (26, 27). Other femoral stem types may affect the femoral anteversion after implantation.

In conclusion, this study found that the AM-CT was the most reliable preoperative reference guide for predicting the PSA when compared to the AM-CT and the AT-3D in DDH patients. The AM-CT at 10 mm height was better able to predict the PSA than the 5 mm height proximal to the base of the lesser trochanter in DDH patients. These findings further underscore the importance of preoperative planning, as it may be challenging to find reliable intraoperative landmarks which can accurately predict the PSA for DDH patients.

## Data Availability

The original contributions presented in the study are included in the article/supplementary material, further inquiries can be directed to the corresponding author/s.
